# From chaos to a new normal—the COVID-19 pandemic as experienced by municipal health and social care providers in Sweden: A qualitative study

**DOI:** 10.1177/20571585221124379

**Published:** 2022-09-28

**Authors:** Maria Tarvis, Kristina Ziegert, Elenita Forsberg, Janicke Andersson, Catharina Gillsjö

**Affiliations:** 1 166447 School of Health and Welfare, Halmstad University, Halmstad, Sweden; 2 School of Health and Sciences, University of Skövde, Skövde, Sweden; 3 College of Nursing, 4260 University of Rhode Island, Kingston, RI, USA

**Keywords:** COVID-19, home care, nursing homes, thematic analysis

## Abstract

When the COVID-19 pandemic began to spread around the world, Swedish municipalities were unprepared. Different guidelines on how to act in relation to the disease varied and protective equipment was lacking. This study aims to describe the experiences of health and social care providers of working at municipality level during the COVID-19 pandemic. A total of 12 assistant nurses, 13 registered nurses, and three physicians were interviewed, individually or in groups, between fall 2020 and spring 2021. The interviews were semi-structured and were analyzed using thematic analysis, utilizing a design following the COREQ-checklist. Three main themes were identified as follows: ‘Initial chaotic situation and uncertainty regarding how to deal with the pandemic’; ‘Continuous changes in organization and work routines’, and ‘Management of the pandemic has become the new normal’. Though health and social care workers eventually managed to embed dealing with COVID-19 as a routine feature of their daily work, municipalities must prepare for future crises.

## Introduction

In Sweden, healthcare is primarily conducted by the 21 regions who have a general responsibility and mandate to provide extensive healthcare activities.^1^ The 290 municipalities conduct healthcare activities within defined areas of responsibility including providing healthcare for older persons and those with disabilities living in nursing homes, and for the bulk of home healthcare provision. Although the municipalities can employ healthcare providers to the competence level of specialist registered nurses, they are not allowed to employ physicians^2^: primary healthcare organizations are responsible for the provision of physicians in municipal healthcare. In order to provide coherent and coordinated healthcare across regional and municipal borders for the most vulnerable patients, a model involving mobile healthcare with physician participation in home healthcare has been implemented to a varied extent in some of Sweden's regions and municipalities.^3–5^

In December 2019, a new disease caused by the SARS-CoV-2 virus, COVID-19, was reported from China, and on 11 March 2020, the World Health Organization declared it a pandemic.^6^ Many countries adopted a strategy of lockdown and closing borders as the COVID-19 pandemic started to spread worldwide, but Sweden took a divergent path, relying mainly on individual responsibility.^7–9^ The main focus of the Swedish strategy, according to recommendations from Sweden's Public Health Agency, was to protect the most vulnerable citizens from the disease and to flatten the curve to prevent overburdening healthcare capacity.^10,11^ Citizens were urged to wash their hands and observe social distancing to avoid becoming infected, and isolation was recommended for persons aged 70 years and over. Over time, this approach to COVID-19 has become a daily routine integrated into the everyday lives of Swedish citizens.

Initially, there was a major shortage of protective equipment in several countries affected by the pandemic^12^ and Sweden was no exception.^8,13^ Opinions varied at the beginning of the pandemic regarding the benefit of face masks in preventing the spread of the COVID-19 virus, and the Public Health Agency stated that wearing face masks in public or in health and social care in the municipalities was not required since there was no clear evidence of their effectiveness.^14^ Face masks were not introduced in municipal health and social care until deaths caused by COVID-19 occurred in nursing homes.^8^

Recent studies show how hospital employees are putting their own health at risk on the front lines of the COVID-19 pandemic.^15–17^ Working under the threat of the pandemic has caused an increased risk of emotional and mental health problems with symptoms such as insomnia, depression, and anxiety, similar to the results of studies performed in nursing homes and home care.^18–20^ It is clear that the COVID-19 pandemic was, and still is, a challenge for health and social care providers worldwide. However, although there are several recent studies examining employees’ experiences of working during the pandemic, the number of studies to understand the situation with COVID-19 in a municipal context is limited. A high infection rate among people receiving home care or living in nursing homes in Sweden^7^ has probably affected employees who work in this context. The aim of the present study was to explore health and social care providers’ experiences of working in the municipality during the COVID-19 pandemic, and how they integrated the pandemic into the daily routines.

## Materials and methods

The study has a qualitative exploratory design^21^ and was carried out in two rural municipalities in western Sweden between September 2020 and April 2021. Data were collected through individual interviews and focus group interviews. Focus group interviews were chosen because of the benefit of participants’ interaction, which enriches and deepens the information.^22^ The thematic analysis developed by Braun and Clarke^23^ was used to identify scenarios and develop themes in order to describe health and social care providers’ experiences of the COVID-19 pandemic at the municipal level. The study was performed in accordance with the Consolidated Criteria for Reporting Qualitative Research (COREQ) checklist^24^ to enhance quality and transparency.

### Participants and recruitment

The participants in the study were recruited based on the following criteria: 1) registered nurses (RN) employed in the municipality, with at least six months of experience of municipal healthcare, 2) assistant nurses (AN) employed in the municipality, with a minimum of six months of experience of working in home service or nursing homes, and 3) physicians working in mobile healthcare (MHC), with at least six months of experience of collaboration with municipal health and social care providers. The researchers first received permission from the heads of the clinical units. Health and social care providers who met the inclusion criteria were identified and asked by their immediate superior if they were interested in participating. A total of 28 health and social care providers from two municipalities participated in the study (see Table 1). The participants received written information about the study that emphasized the voluntary nature of participation and their right to withdraw at any stage. All study participants signed an informed consent before the interview, and there were no withdrawals from participation during the study.

**Table 1.** Characteristics of the participants.

Participants	28 (total)
Female	27
Male	1
Age (years)	
Mean	49.1
Median	50
Range	23–64
Role/Job category	
Assistant nurses	12
Registered nurses	13
Physicians	3
Working experience (years)	
Mean	12
Median	14
Range	2–24
Focus groups	5 (total)
Assistant nurses	10 (2, 3, and 5 participants)
Registered nurses	9 (3 and 6 participants)
Individual interviews	9 (total)
Assistant nurses	2
Registered nurses	4
Physicians	3

### Data collection

This study is part of a larger project studying health and social care providers’ experiences of working in home-based care. A total of six focus group interviews, three with RNs and three with ANs, were conducted by the first author in September and October 2020 at the participants’ workplace, before the second wave of the pandemic hit Sweden. The number of participants in the focus groups was in the range of 2–6, and the interviews lasted 49–108 minutes. During each focus group interview, one of the co-authors listened via Zoom and, if needed, added follow-up questions to deepen the dialogue. The co-author was presented, and the purpose of participation was explained to the participants before each interview. The focus group interviews and the individual interviews with the physicians were based on a semi-structured interview guide developed by the first author, and the questions were pilot tested in the first focus group interview. Only the last question in the interview guide referred to the COVID-19 pandemic. One focus group interview (n = 5) with RNs did not include the question regarding experiences related to the pandemic and the participants were asked if they were willing to participate in individual interviews. Four of them agreed and were interviewed in December 2020 to April 2021 after the second wave of the pandemic. The interviews were 7–17 minutes long. Due to their difficulty in being absent during working hours, the physicians were interviewed individually. The interviews were conducted in October to December 2020 before the second wave of the pandemic and lasted 19–20 minutes. The individual interviews with RNs and physicians were performed by the first author via mobile phone. To collect varying perspectives regarding the subject matter, two ANs were recruited for individual interviews, which were carried out by the first author in their homes in June 2021. The interviews lasted 17–30 minutes. The main question in all interviews for this study was: Would you please describe your experiences of working during the COVID-19 pandemic? Follow-up questions were used to elaborate on their responses. All interviews were audio recorded and transcribed verbatim by the first author.

### Data analysis

Due to the ongoing COVID-19 pandemic, collaboration between the authors was conducted via internet meetings. A semantic approach was used for data analysis based on the thematic analyses devised by Braun and Clarke^23^ (see Table 2).

**Table 2.** Thematic analysis process.

Phase 1: familiarization	Each of the five co-authors familiarized themselves with all the transcriptions and their colleagues’ field notes. (All authors)
Phase 2: coding	Meaning units were identified and coded, then they were sorted into initial scenarios (i.e. experiences of the COVID-19 pandemic in a work context). (All authors)
Phase 3: searching for themes	A thematic map was created. Each scenario was sorted and categorized into three preliminary main themes. (All authors)
Phase 4: reviewing themes	The preliminary themes in the thematic map were contrasted and refined into three main themes and seven sub-themes. (Author nos. 1, 2, and 5)
Phase 5: defining and naming	Intersubjective agreement regarding the defining and labelling of themes was reached among the co-authors. The themes were finally defined and labelled. (All authors)
Phase 6: producing the report	The analysis report was written. (Author nos. 1 and 5)

### Ethical considerations

Ethical approval was granted by the Swedish Ethical Review Authority (2019-06383), and the study was conducted in accordance with the Declaration of Helsinki^25^ and the Ethical Review Act.^26^ Participants were given both oral and written information about the aim of the study and the procedure. They were informed that participation is voluntary, of their right to withdraw at any time, and that personal data would be handled in accordance with the General Data Protection Regulation (GDPR).^27^ To ensure confidentiality, all personal data were decoded and stored in a security cabinet, apart from the encoding list, and only researchers in the project had access to the cabinet. Oral and written consent to participate in the study was obtained from each participant.

### Pre-understanding

The first author worked in municipal healthcare as a registered nurse before and during the data collection. Before the interviews and focus groups, the author's working position was revealed to the informants. The first author's understanding of working in municipal health and social care during the COVID-19 pandemic was critically considered throughout the study.^28^ None of the other four authors worked in health and social care during the study.

## Results

The aim of the study was to explore health and social care providers’ experiences of working in the municipality during the COVID-19 pandemic, and how they integrated the pandemic into the daily routines. The identified scenarios in the interviews were further analyzed into three themes and seven subthemes (see Table 3), illustrated by quotes.

**Table 3.** Themes and sub-themes and the underlying scenarios and scenario characteristics.

Scenarios	Scenario characteristics	Sub-theme	Theme
A chaotic situation	Guidelines changing on a daily basis Limited facilities in workplaces Maintaining social distancing a challenge	Limitations in knowledge, equipment, and guidelines	1: Initial chaotic situation and uncertainty regarding how to deal with the pandemic
Fear and frustration	Fear of being infected and of transmitting infection to others Fear of dying Frustration related to limited access to protective equipment	The pandemic creates fear and frustration	
Incorrect actions	Misinterpretation of unclear guidelines Mistrusting the protective equipment	Increased risk of incorrect actions	
Covid-19—the highest priority	Reorganizing daily work to prioritize the disease Flexibility—a prerequisite	Continuous adjustment of health and social care	2: Continuous changes in organization and work routines
The individual—the second priority	Patients not having individual assessments Patients left to die alone Home care services cancelled	The pandemic overshadows individual needs	
Incorporating COVID-19 into daily routines	Getting used to the situation Protective equipment that can be trusted	Learning to handle the situation	3: Management of the pandemic has become the new normal
Need for the team	Supporting each other in the team Attention to needs MHC is an important member of the team	Dealing with the pandemic as a team	

### Theme 1: Initial chaotic situation and uncertainty regarding how to deal with the pandemic

This theme describes the chaos that was experienced in the initial phase of the COVID-19 pandemic. There was a lack of knowledge regarding how to deal with the disease in practice, protective equipment resources were limited, and guidelines were vague and changed frequently. This resulted in a situation described as ‘chaotic’, a time marked by feelings of fear, frustration, and uncertainty, and the potential risk of incorrect actions being taken.

### Limitations in knowledge, equipment, and guidelines

Lack of knowledge about the disease, the shortfalls in protective equipment, and frequently changing guidelines in the initial phase of the pandemic created uncertainty. The participants described an initial knowledge deficit, or contradictory knowledge, regarding the transmission of COVID-19. The uncertainty regarding how to deal with the disease, especially in relation to routines and protective equipment, were obvious and reflected in the sudden changes of guidelines in local, regional, and national work settings. The situation was described by one AN:

‘I think it was complete chaos… It was actually terrible.’ (AN No. 2, focus group)

The changing guidelines, which could be modified from one day to the next, were to some extent seen as more stressful than the pandemic itself.

The participants described how the guidelines initially required the use of face shields and face masks only when a patient was either suspected of or confirmed as being infected with COVID-19. They had to provide health and social care to several patients every day without knowing if they were encountering infected persons, wearing only the traditionally required protective equipment of gloves and aprons, which reinforced the feeling of chaos and uncertainty:

‘We would not have face masks, because that was the rule… we would keep our distance… we would use soap and water and surface disinfection and hand sanitizer. That's what was supposed to help us.’ (AN No. 12, individual interview)

The participants described the guidelines drawn up regarding personal hygiene and work clothes to prevent the spread of COVID-19. Workplace facilities were limited and made it difficult and sometimes impossible to take a shower, change, or wash contaminated clothes:

‘And then the employer expects that we should maintain a certain level that they cannot uphold themselves, for example, washing clothes and so on.’ (RN No. 3, focus group)

Enforcing social distancing with both colleagues and patients was described as challenging. Premises were often cramped, and some patients did not fully understand that there was a pandemic and the importance of social distancing in avoiding transmission. Inventiveness was required and participants described how ANs put notes on the doors of patients with dementia as a reminder about the disease, hoping it would help them to understand the need to remain in their own rooms.

### The pandemic creates fear and frustration

The initial chaotic pandemic situation during spring 2020 created irritation, stress, fear, and frustration. Participants described how so much was unclear in the beginning and questions were raised regarding how it all might work out and the risk of dying. They described a frustrating situation, with fears of becoming infected and anxiety regarding accidentally infecting their own families or patients, especially vulnerable patients:

‘You never know… am I carrying it with me or not?… That's what I’ve been thinking about for a very long time… will I transmit it to someone or not?’ (AN No. 9, focus group)

The participants described how this uncertainty and the fear of either catching the disease themselves or transferring it to patients in the chaotic situation increased their use of materials such as hand sanitizer:

‘I know that the first weekend I finished seven pump bottles of hand sanitizer… I counted them since I had them on a separate table…’ (AN No. 11, individual interview)

Information meetings with the region's infection control unit, about the disease and how to manage it to prevent and reduce transmission, was described as useful in diminishing participants’ fears, frustrations, and uncertainty. The information focused on social distancing, hand washing, and disinfection of hands and surfaces. One AN said that the information that it was possible to kill the virus using soap and water reduced fear and inspired confidence in coping with the situation, given earlier experiences of infections with resistant bacteria. However, a major difference at this time was that the regular wearing of face masks was not permitted for daily work. However, even when the guidelines changed and the use of face masks and face shields on a daily basis was allowed, feelings of fear and frustration in relation to transmission of the disease continued since so little protective equipment was actually delivered:

‘So, I went down to the basement to look at this box… Then I got worried because I thought, if we were to experience a mass outbreak of infection…’ (AN No. 12, individual interview)

The AN realized that it would not be enough in the event of a large COVID-19 outbreak.

Inadequate knowledge and limitations on access to protective equipment, together with frequently changing guidelines, added to the fear and frustration, not only for the participants but also for patients and their next of kin. These fears and frustrations regarding COVID-19 and its transmission manifested in different ways. Participants described debates over whether ANs in home care services should be allowed to buy groceries for their patients, due to the risk of spreading COVID-19. RNs were to some extent hindered from seeing physicians in primary healthcare centers due to the centers’ fear of allowing RNs onto their premises. One of the RNs described how this conveyed the feeling of being infected and a carrier of the plague. Participants also stated that work premises often were cramped, and maintaining social distance was not easy. The Public Health Agency directive, that even if a person in the family had COVID-19 other family members had to go to work, was challenging under these circumstances. One AN with a family member infected with COVID-19 at home described a situation at work:

‘So, I said this at work… with the consequence that I was totally outcast… Oh, and they said that I should have lied and said that I should have called in sick.’ (AN No. 12, individual interview)

Co-workers’ fears and frustration and colleagues’ approach to the current guidelines led to an experience of being shunned by the rest of the group.

### Increased risk of incorrect actions

The limited knowledge about COVID-19, the deficit in protective equipment, contradictory information, and frequently changing guidelines left space for individual interpretation and decisions in relation to the prevailing guidelines with the consequent risk of incorrect actions. The participants described misunderstandings regarding how and when to perform basic hygiene routines and how and when to use face shields and face masks. Guidelines were not always respected or taken seriously, especially with regard to how and when to use protective equipment. Using face shields and face masks was seen as complicated, uncomfortable, and frustrating, and situations were described in which they were used incorrectly or not at all:

‘… face shields have not worked, they just break all the time and… Yes, I must honestly say that sometimes I have just thrown it away and worked without it.’ (AN No. 10, focus group)

The participants’ feelings in relation to protective equipment varied. Some reported being grateful and happy when face shields were introduced into health and social care, feeling convinced that the equipment protected not only against COVID-19 but also against other viruses and bacteria. At the same time, participants said that their earlier dissatisfaction and lack of confidence resulted in uncertainty regarding the effectiveness of face shields in preventing infection. Besides being uncomfortable and complicated to use, the face shields were described as not fitting properly and were not trusted to prevent the disease because they were open on all sides:

‘If you sneeze, it's all around… It feels like it's just for show, to be honest.’ (AN No. 10, focus group)

The doubts regarding the protection afforded by face shields reduced the motivation to use them and increased the risk of incorrect actions. One RN stated:

‘So, the most important thing is… to protect the patient, it is actually the face mask that is best. Not the face shields but the face masks are… the best to protect the patient.’ (RN No. 13, focus group)

The participants wanted to have face masks to protect the patients and to feel safe but did not have access to them.

### Theme 2: Continuous changes in organization and work routines

This theme describes how the COVID-19 pandemic affected municipal health and social care on both an organizational and individual level. The increased workload connected to the pandemic resulted in continuous adjustments to daily work routines where tasks linked to the disease were prioritized over other assignments and overshadowed individual needs.

### Continuous adjustment of health and social care

The COVID-19 pandemic required continuous adjustments and reprioritizations in the provision of health and social care. The whole situation was a challenge for the organization and demanded an increased intensity in daily work as the workload escalated. The participants described situations in which colleagues had to stay at home or go home at short notice due to symptoms that could be interpreted as those of COVID-19. This required flexibility when working tasks had to be reorganized or cancelled due to pandemic-related activities. Frequently testing patients for the disease, conducting COVID-19 contact tracing, and answering phone calls from anxious relatives, as well as providing health and social care to infected patients, was challenging and affected normal work routines.

The weekly meetings between RNs and ANs to discuss patients were canceled or held by phone. Testing for COVID-19, to prevent the spread of the infection, was more important than most of the other tasks and had to be prioritized, especially when someone was exhibiting symptoms associated with the disease. The need for reprioritizations and sudden adjustments was described by one RN:

‘But you had to postpone many things… because of the COVID patients, I mean suddenly there is an infection tracing required and then you have to let go of everything else.’ (RN 8, individual interview)

The participants described their relief and their appreciation of the special teams that were organized in the municipalities to provide health and social care for patients with COVID-19 and associated tasks such as testing for and the tracing of infection in ordinary homes and in nursing homes. The teams helped to ease the workload and reduce the risk of disease transmission.

The participants said that access to physicians at the primary healthcare centers became more difficult because of the pandemic. At the same time, there was an increased need for physicians regarding the prescription of tests for patients with suspected COVID-19, assessments, and prescribing medications. Additional physicians were assigned to the MHC scheme and during summer 2020 the ongoing project became permanent:

‘It was almost like the pandemic… quite clearly highlighted that home healthcare must be a priority.’ (Physician No. 3, individual interview)

### The pandemic overshadows individual needs

The participants described several different areas and situations in which the pandemic overshadowed individual needs. They noted that reports regarding deficiencies and inequalities in the provision of health and social care to older persons infected with COVID-19 living in ordinary homes or in nursing homes in Sweden were widespread in all forms of media—newspapers, television, and online media. The participants agreed to some extent with these reports and recognized the risk of not meeting individual needs during the pandemic, but also disagreed with allegations that the pandemic was overshadowing individual needs. They described reports in the media focusing on patients not receiving an individual assessment from a physician in person; instead, the assessment was based on a telephone conversation between the physician and RN. They highlighted the importance of remembering that not all patients diagnosed with COVID-19 required a visit from a physician:

‘All the criticism that exists from the outside, and that you… don't see the needs in every human being… I think… we really did. Each person has received an individual assessment. However, not everyone has been medically assessed by a physician because there is no need for that.’ (RN No. 8, individual interview)

Situations reported in the media where patients receiving palliative care were left in nursing homes without access to oxygen and other forms of treatment instead of being transferred to a hospital were experienced by participants. Patients in long-term care setting were left alone to die with no one to hold their hand. Participants described the upset caused by witnessing patients gasp for air: in some cases, the guidelines were ignored, and patients were sent to hospital despite the regulations but in accordance with ethical and professional responsibilities.

Participants also described COVID-19's major impact on the provision of in-home healthcare services. During the first months of the pandemic, several patients canceled their home care services since they or members of their families were afraid of contracting COVID-19. This fear was not only connected to the disease itself but also to the fact that health and social care providers did not wear protective equipment, such as face masks and face shields. At the same time, there were patients who had no other choice but to accept assistance. The participants felt they were viewed as bearers of death and described how they did their best to follow the guidelines:

‘We have been given certain guidelines and that is the only thing we have been able to do… do what the manager and what the municipality has said we should do. And that's what we have had to refer to.’ (AN No. 10, focus group)

The lack of protective equipment created an uncomfortable situation whereby patients and their families regarded the participants as a potential threat.

### Theme 3: Management of the pandemic has become the new normal

This theme describes how managing the COVID-19 pandemic over time has become the new normal in the work setting. The participants learned to manage the situation in relation to patients, their families, and themselves. Professional responsibility, teamwork, and support from co-workers and managers were required to learn how to handle the situation and deal with the pandemic as a team.

### Learning to handle the situation

When knowledge about COVID-19 improved and the guidelines became firm and consistent, handling the pandemic became the new normal in the work setting. Staff learned to handle the situation in relation to patients, their families, and within their profession. The special teams helping out with infected patients, testing, and contact tracing were withdrawn after the first wave of the pandemic. Assessment of patients’ symptoms, performing COVID-19 testing, and tracking transmission of the disease were now an ordinary part of the RNs’ daily work. Respite care recommenced and the patients had to be tested for COVID-19 both before and after each visit, which was considered a demanding task. At the same time, the testing had become a routine:

‘It becomes easier and easier when you learn how to handle it… yes… So, it works well, I think.’ (RN No. 7, individual interview)

According to the Communicable Diseases Act, anyone with symptoms that could indicate COVID-19 is required to stay at home as a matter of personal responsibility, even when those symptoms are mild or do not exactly match those commonly associated with COVID-19. The participants described how unclear symptoms could sometimes be stressful and lead to uncertainty regarding the need to report sick and stay at home. The obligation to stay at home when having symptoms also caused staff shortages, and participants stated that they had never before experienced absence rates as was the case during the pandemic. They described their worries and how difficult it was constantly thinking of the risk of spreading the infection to someone. Over time, they learned to deal with these feelings, especially once they were receiving unlimited quantities of protective equipment that they felt could be trusted:

‘If you follow the existing recommendation… you must believe that you have done the right thing.’ (AN No. 8, focus group)

### Dealing with the pandemic as a team

The increased workload was described as stressful, with the disease affecting daily work, and support from colleagues and managers was crucial to deal with the pandemic's effects on the provision of health and social care. COVID-19 became incorporated as the new normal, a situation the team had to address in their daily work. Close teamwork between colleagues and other professions was considered critical, to support each other rather than being put on hold when calling or having to drive long distances to meet up. ANs came to be considered as an extra arm, trusted by RNs to monitor patients and get in touch with RNs when needed. Team members were described as having become closely knitted together, working under the threat of the disease strengthened collaboration. Support from colleagues was fundamental in coping with the stressful situation, but it was also important to receive support from immediate superiors:

‘The manager was fantastic in this; it was just like “What do you need? Call me, tell me what you want, tell me what you need, I will come.”’ (AN No. 11, individual interview)

The participants said close teamwork was required to handle the situation and create new ways of working to deal with the pandemic and incorporate the measures required to combat it into their daily work.

MHC involvement in the team was seen as important. One RN stated that MHC physicians had a different attitude toward the disease, compared to some of the physicians at the health centers who were seen as being terrified and therefore unwilling to visit infected patients:

‘The home healthcare physician is the one who has absolutely done most of the work with those who have had COVID with severe symptoms… The primary healthcare centers have not had that approach at all… they have been terrified… They have had a very strange attitude.’ (RN No. 8, individual interview)

The willingness of the MHC physicians to visit patients who were infected with COVID-19 and prescribe medical examinations and treatments was considered crucial to handling these patients at home.

## Discussion

The present study reveals a range of scenarios leading from an initially chaotic situation of not knowing how to deal with the COVID-19 pandemic in daily work, through continuous changes in work routines, to a new normal where managing the pandemic became routine on a daily basis. When municipal health and social care was required to respond to COVID-19, it was clear that neither the health and social care organizations nor the providers were prepared for managing a situation as extreme as a pandemic. The need for clarity is highlighted by the findings in the current study, showing that unclear or contradictory guidelines were obstacles to be surmounted while trying to achieve a new balance in the changed context of the pandemic. This finding is in line with the results of a recent study, which describes the experience of insecurity related to the flow of contradictory information from management.^29^ Limited knowledge and the continuous changes in guidelines during the pandemic prevented health and social care providers from feeling confident and safe in work situations. Under normal circumstances, guidelines are the foundation for the provision of health and social care, and firm and consistent guidelines are crucial to prevent fear and confusion in crisis situations.^30,31^

The participants in the present study experienced difficulties in finding a balance and upholding their professional roles as providers of good health and social care. They described how they felt unsafe not knowing if they would be infected or even die from the disease, lacking both adequate facilities and protective equipment. They were viewed as potential carriers of COVID-19 and were sometimes even associated with death. The media reported on deficiencies in health and social care in ordinary homes and nursing homes in Sweden, and about how patients were left to die without individual assessment or treatment. Experiencing stigma is common among caregivers who care for patients during epidemics and pandemic outbreaks,^32^ and exaggerated pandemic reports from the media may contribute to the feeling of being stigmatized.^29^ This finding is consistent with a study of nurses who worked with infected patients during the Middle East Respiratory Syndrome outbreaks, which perceived themselves as ‘vermin’ carrying the deadly disease due to the intense media coverage.^33^ This illustrates how the media can influence the image of health and social care providers, which is particularly important to consider during a period when they are already vulnerable such as during a pandemic.

The findings in the current study emphasize that closeness to colleagues was crucial in terms of providing mutual supporting during the crisis, which is also consistent with results from previous studies.^29,34–37^ Working together during difficult circumstances not only reduces stress and anxiety, it also creates strong bonds among co-workers.^29,30,34,38,39^ The participants in the current study described how working closely together strengthened team camaraderie and how they cooperated to integrate the pandemic into their daily work. They trusted their colleagues’ competence and appreciated the support they gave each other. The health and social care providers needed to deal with the initial chaotic situation and repeatedly negotiate, adjust, and organize their way of working to embed and integrate the pandemic as a new aspect of their daily work.

### Strengths and limitations

The interviews in this study were conducted after the first and second waves of the COVID-19 pandemic, which can be considered a strength since some participants interviewed after the first wave had no personal experience of the disease. Until the summer of 2020, when the ongoing physician participation in home healthcare projects became permanent in the region, only a few physicians were involved. As physicians are a critical aspect of healthcare, the low number of only three participants may have affected the study results. The variation in data collection methods, using both individual and group interviews, may have affected the depth of data collected. However, this potential difference in data depth was addressed in the group interviews using targeted questions to ensure that everyone shared their experiences. Although the individual interviews proved to be relatively short, the data collected were consistent with the data from the focus group interviews. The thematic analysis of Brown and Clarke was chosen for the data analysis because of its flexibility.^40^ Although the method allows virtually any type of qualitative data for analysis and both large or small datasets, it adds complexity and richness to the presentation of data. Thick description and verbatim quoting were used to achieve transferability, and the close collaboration between the authors during both data collection and analysis in iterative cycles strengthens the trustworthiness of the study's findings.^28^

## Conclusion

The study's findings reveal an appreciable vulnerability in municipal health and social care in Sweden. The initial unpreparedness with continuous changes of guidelines, and work routines resulted in uncertainty, fear, and frustration. This highlights the importance of developing contingency plans and train different scenarios to prepare for future crisis in the organization. This is a prerequisite to be able to provide good and secure health and social care in ways that preserve and promote health and wellbeing during challenging circumstances as a pandemic.
